# An Experimental Study of Clogging Recovery Measures for Ceramic Permeable Bricks

**DOI:** 10.3390/ma14143904

**Published:** 2021-07-13

**Authors:** Zizeng Lin, Hai Yang, Huiming Chen

**Affiliations:** 1 College of Civil Engineering, Nanjing Forestry University, Nanjing 210037, China; chm@njfu.edu.cn; 2 Sichuan Road & Bridge (Group) Corporation LTD., Chengdu 610093, China; yang.hai@srbg.no

**Keywords:** ceramic permeable brick, maintenance recovery, clogging

## Abstract

To explore the best clogging restoration measures for ceramic permeable bricks, ceramic permeable bricks were accurately clogged using a self-designed device by controlling the permeability, and different technical measures were adopted to restore the permeability. Then, the recovery effect, operating parameters and pore change inside the bricks using pressure washing were further discussed. The results showed that pressure washing was the best recovery measure, the joint methods was not recommended due to performance to price ratio. It was necessary to conduct pressure washing in relatively moist conditions, increase the cleaning frequency or prolong the cleaning time in the case of no serious blockage. Hydraulic cleaning can not only increase isolated pores but also remove the trapped solid particles, and increase the proportion of connected pores and dredges through water channels. This research offers some reference for the daily maintenance of permeable bricks.

## 1. Introduction

In recent years, permeable brick pavements have become one of the most frequently used low impact development (LID) techniques [1,2,3]. This infiltration-based technology is comprised of structural layers with relatively high porosity to allow rainwater to pass through its surface brick and underlying structure. Permeable brick systems play a significant role in the hydrological effect and reduction in rainwater pollution [4,5,6].

Newly installed permeable brick pavements provide sufficient permeability to reduce runoff volume and delay peak flows during frequent rainfall events. However, the permeability coefficient of permeable brick declines with time as sediment and debris clog pore spaces. Many factors, such as the particle distribution of the sediment in runoff, the pore size distribution of the void spaces, and the size of the clogging particles relative to the pore size [7], have been suggested to influence clogging and hamper hydraulic functionality. The removal of particulates by permeable bricks is the most significant factor in the occurrence of blockages. Paradoxically, a permeable brick system exhibits favorable decontamination potential, highlighted by the removal of total suspended solids (TSS) from permeable brick. Li et al. [8] revealed that the average removal efficiency of TSS was approximately 90.0% for six commonly used permeable pavement materials because of physical interception. Zizeng et al. [2] also found that the physical interception of the surface layer played a critical role in the SS filtration process, and the uniform and dense pore distribution was instrumental in the retention of particulates. Due to the high removal capacity of TSS in urban runoff, sediment is captured in the voids within the pavement material, and physical blockage occurs [9], which is why clogging has been defined as the accumulation of silt within the brick due to sedimentation [10]. Therefore, clogging has been found to be greatly affected by years of service [11]. With the increase in the retention of solid particles, especially finer particles [12], permeable bricks are gradually blocked, and their lifespan basically terminates [13]. Then, effective recovery techniques and technical procedures are needed to restore the hydraulic functionality and water control benefits [14].

At present, research on rehabilitation measures for pavement permeability mainly focuses on permeable concrete (PC), permeable interlocking concrete pavers (PICP), and porous asphalt (PA), while there is little research on permeable bricks (PB). Permeable bricks, especially ceramic permeable bricks, are commonly used in the Chinese market because of their compact structure and good hydrological performance. Therefore, it is necessary to study the restoration measures of permeable bricks drawing on relevant technologies. Currently, mechanical street sweeping, vacuum suction or high-pressure water washing are the three main technologies used to restore the permeability of PC, PICP, and PA. Table 1 summarizes the recovery measures and effects of different permeable pavements in recent years [15,16,17,18,19,20]. As seen from Table 1, permeable pavements with different pavement ages had different sensitivities to the restoration measures, and there was a significant difference in the recovery effects of the same measure for different permeable pavements. For instance, in the PC recovery research of Manahiloh [20], the restoration effect of vacuum treatment was partial, while porosity recovered from 26% to 29%; however, in the PA recovery research of Winston [17], the post-maintenance permeability coefficient of was 3.5 times higher than that pre-maintenance using vacuum, while that of pressure washing was 360 times higher. Hence, it is meaningful to conduct a detailed evaluation and comparison of the restoration effects of the three techniques and their combination on permeable bricks. Then, the specific operating parameters should be further investigated to improve the adaptability of the technology. The pore types, characteristics, and parameters are of great significance for the study of the clogging mechanism of the bricks [21]. Conversely, the main mechanism of recovery may be explored by investigating the pore change in the recovery process; obviously, it is necessary to study the pore change inside the brick.

In the present study, ceramic permeable bricks were accurately clogged using a self-designed device by controlling the permeability coefficient. After clogging, different technical measures were adopted to restore the permeable brick, and the permeability coefficient was measured to explore the best clogging restoration measures of ceramic permeable bricks. Then, the specific operating parameters using pressure washing were further investigated, and X-ray Computerized Tomography (CT) was used to obtain the pore size and connection state. Data such as the porosity and matrix skeleton ratio were also obtained to quantitatively determine the pore distribution, and the process of maintenance recovery of the permeable brick was further discussed. The purpose of this research was to explore the suitability and effectiveness of maintenance measures for restoring the permeability of permeable bricks.

## 2. Materials and Method

### 2.1. Permeable Bricks

**Figure 1:**
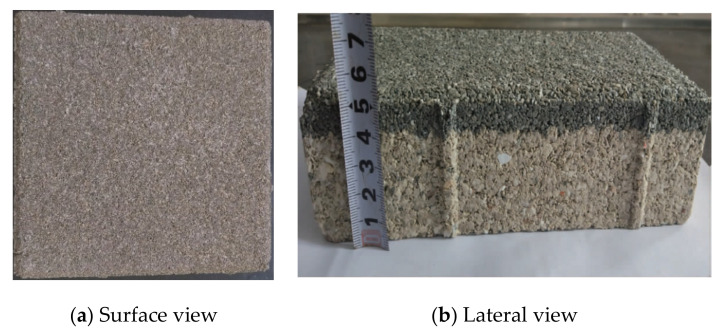
Ceramic permeable brick.

**Figure 2:**
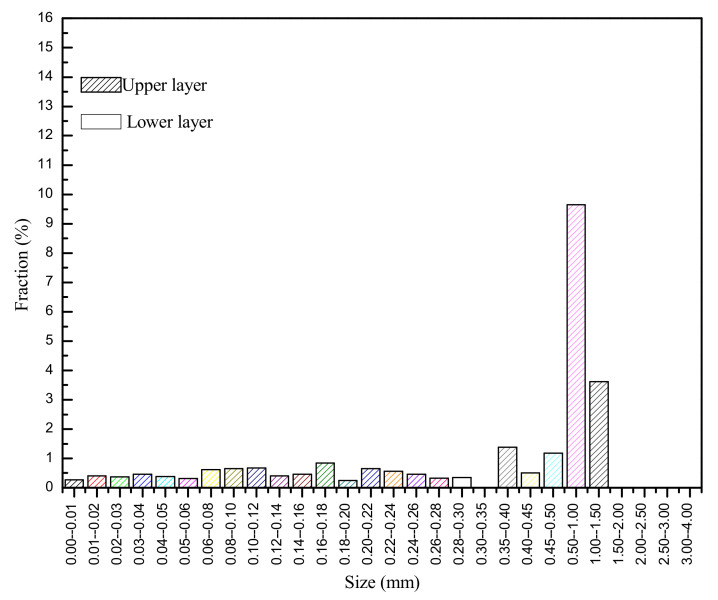
Pore size distributions of the upper and lower layers.

The ceramic permeable bricks measured 20 cm × 20 cm × 5.5 cm and were purchased from the Youbang Building Materials Co., Ltd., Yixing, China. The ceramic permeable bricks were sintered at 1200 °C using waste ceramics as the main raw material. They are mainly used in the construction of permeable pavements in city parks, squares, parking lots, and residential areas. Similar to all permeable bricks in the Chinese market at present, ceramic bricks can be divided into two layers in a common configuration (Figure 1). The upper layer has a thickness of approximately 1.0 cm with particles having a relatively small diameter, and the lower layer is approximately 4.5 cm. The pore size distributions of the upper and lower layers are shown in Figure 2.

### 2.2. Original Filtration Suspension

Previous monitoring found that the concentration of TSS in initial rainwater near a busy traffic road in Nanjing was 800 mg/L−1314 mg/L; therefore, the original suspension with 1000 mg/L was selected for the simulation experiment in this study. The particle size distribution of the original TSS suspension refers to the research results of Duncan [22] and Zuo [23]. Duncan’s study of sediment particle size in rainwater found that particles smaller than 85 μm accounted for approximately 90%. Zuo investigated the particle size of highway runoff in Nanjing and found that most particles were in the range of 0.45–20 μm in runoff, accounting for 37.5% of the total particles.

**Figure 3:**
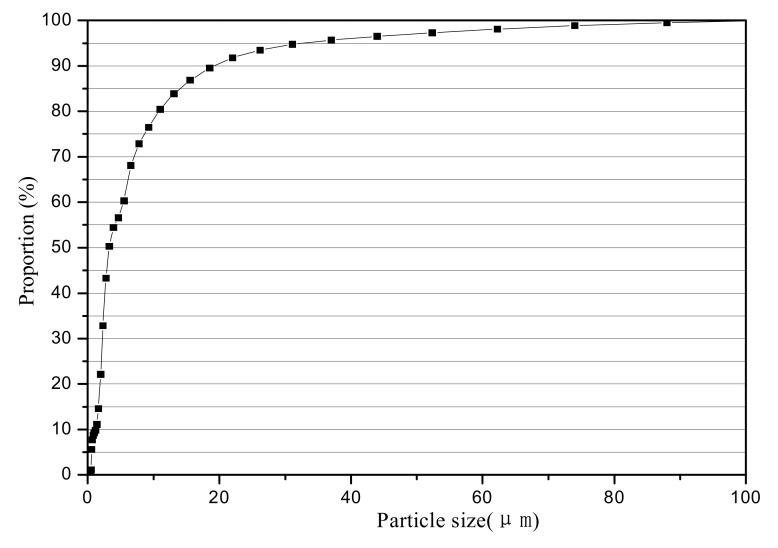
Particle distribution of the original TSS suspension.

In this study, kaolin was first dried and sieved using a 140 mesh sieve to remove particles with diameters larger than 0.106 mm, and then, kaolin with different particles was obtained by filtering through sieves with different diameters. Particles of different sizes were uniformly mixed as sediment samples at a fixed mass ratio in rainwater. Finally, the original suspension with a 1000 mg/L concentration whose particle distribution was more consistent with the actual rainwater was obtained. Surface active agent was added to rainwater to disperse the particles, then the particle distribution was measured using a laser particle size analyzer, shown in Figure 3. The results presented in Figure 3 show the size distribution of particles with diameters less than 74 μm, which accounted for approximately 98%, of which 0.45–20 μm was the dominant particle size comprising 53%. The particle size distribution was basically consistent with the formation of particulate matter in rainwater.

### 2.3. The Process of Blockage

**Figure 4:**
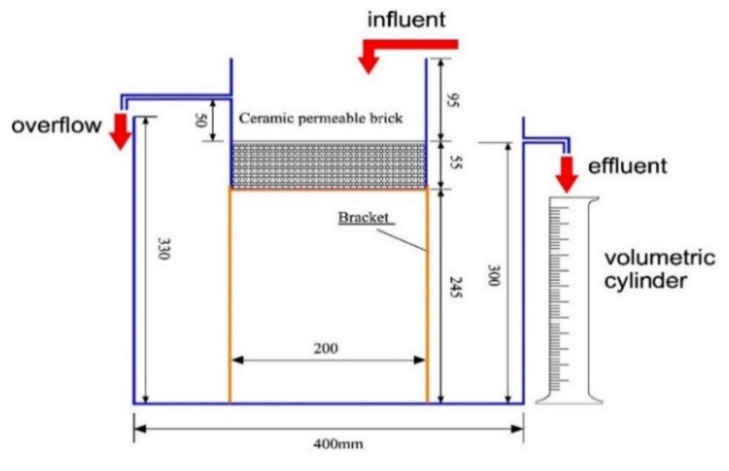
The experimental apparatus.

To accurately control the extent of clogging, a classical device was designed to cope with the dimensions of bricks according to the Chinese national standard of permeable paving bricks and permeable paving flags (GB/T 25993-2010) to achieve precise control of the permeability coefficient during the clogging process (Figure 4). Precise blockage was achieved by filtering a simulated TSS suspension with a 1000 mg/L concentration through the permeable brick placed in the device, while the permeability coefficient was measured using the constant head method, which followed Darcy’s law and can be expressed by the following formula:

$$K=\frac{VL}{AHT}$$

where *K* is the permeability coefficient, *V* is the water volume, *L* is the height of the brick, 5.5 cm, *A* is the horizontal area of the brick, 400 cm^2^, *H* is the water level difference from the upper surface of the brick to the bottom of the overflow pipe, 5.0 cm, and *T* is the duration of the experiment.

Three degrees of clogging, low, medium and high, were designed to filter 5 L, 10 L, and 20 L suspension in our study, respectively. In this case, the permeability coefficients of low, medium, and high degrees of blockage are equal to 78.7%, 46.1%, and 23.4% of not clogged bricks permeability coefficients, respectively.

### 2.4. Experimental Cleaning Scheme

Four factors, the maintenance methods, moist state, degree of clogging, and cleaning duration, were considered in the recovery experiments, and the detailed recovery experimental scheme was designed as shown in Table 2 according to the four factors. In the cleaning scheme, item 6–12 was used to compare the recovery effects of different measures to investigate the appropriate technique. Then specific operating conditions for the best method (P) were investigated, the recovery effects of moisture state were compared using experimental items 6 and 13, and items 1–6 were used to compare the influence of cleaning duration under different degrees of blockage.

The clogging experiment was carried out using the designed device before the recovery experiment. After the clogging experiment, different technical measures were adopted to restore the permeable brick, and the permeability coefficient K after restoration was determined. The restoration effect is calculated according to the following formula:

$$\eta =\frac{K}{K_{0}}\times 100\%$$

where η is the recovery rate (%), K is the permeability coefficient of clogged brick, and $K_{0}$ is the permeability coefficient of unclogged new bricks.

### 2.5. Test Methods

According to the Chinese National Standard Methods (SEPA of China 2002), the TSS was determined by the gravimetric method (GB 11901-89). According to the linear transverse winding test of the Standard Test Method for Microscopical Determination of Parameters of the Air-Void System in Hardened Concrete (ASTM C457), the pore size distribution of the ceramic brick were measured using an air void analyzer (Rapid air 457, Germany).The particle size distribution of the sediment in the rainwater was measured using a laser particle size analyzer (Mastersizer 3000, England), and surface active agent (Nonidet P40) was added to rainwater to disperse the particles to monitor the distribution.

Manual surface cleaning using a tube brush cleaned the surface of permeable brick for three minutes. Vacuum cleaning was conducted using a vacuum pump with a negative pressure of 30 kPa, and the vacuuming process also lasted three minutes. Pressure washing was conducted using a mobile wash truck with a pressure of 0.3 MPa for three minutes, while the water gun was at an angle of 45 degrees from the brick.

The pore size distribution of the ceramic bricks different cleaning processes were measured using the CT method. CT scanning and image processing were carried out using a nanoVoxel3000 CT scanner. Then, VoxelStudio Recon 3D reconstruction software was used to restore the samples, and Avizo software was used to calculate and extract sample information. To avoid error caused by a single brick measurement, eight bricks were repeatedly tested in the process of blockage and recovery, and the average value was used as the basis for analysis.

## 3. Results and Discussion

### 3.1. Recovery Effects of Different Measures

**Figure 5:**
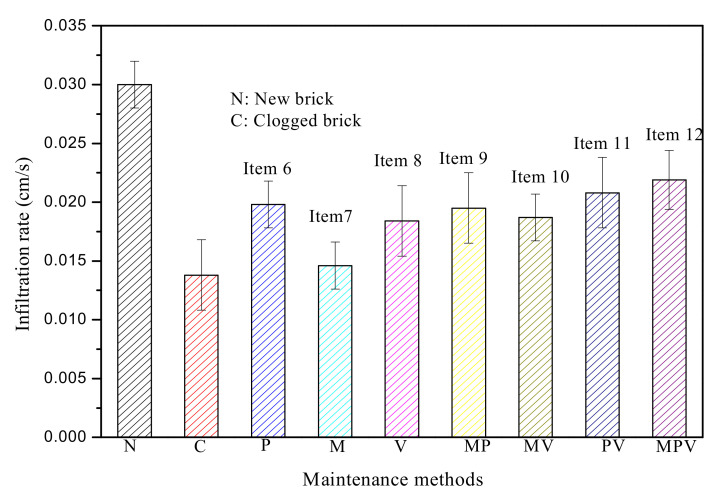
The recovery effects of different maintenance methods.

To optimize the best clogging restoration measures, single measure, two-combination measures and three-combination recovery measures were compared and discussed in this part. The recovery effects of different measures are shown in Figure 5. As shown in Figure 5, among the single measures, pressure washing had the best recovery effect, followed by vacuum suction, and manual cleaning had the worst recovery effect. For permeable bricks moderately clogged to an initial permeability of 46.0%, the permeability coefficient could be restored to 66.0% of the initial permeability after pressure washing, 61.3% after vacuum suction, and 48.7% after manual cleaning.

Studies have shown that most of the clogged particles were in the shallow location (1.0 cm) on the surface of the ceramic permeable brick during permeable brick blockage, which was mainly determined by the structural configuration of the brick (Figure 2) [21,24]. Pressure washing (P) flushed out the particles that had accumulated on the surface and allowed the clogged particles inside the brick to move energy to come out with the flushing flow, thus increasing its permeability. A study found that the clogged sediment was significantly different under different rainfall intensities, which also meant that a flushing effect could be caused by larger washing intensities, thus reducing the clogging particles [12]. It is speculated that pressure cleaning could remove both surface and internal blockages, and thus, the cleaning effect was the best. The average infiltration rate of ceramic permeable brick using P increased from 0.0138 cm/s to 0.0198 cm/s by an average of 43.5%, it shows that P is a very effective recovery measure. Vacuum suction (V) could only extract clogged particles that were not strongly adhered in the pores [25], and thus, the recovery effect was inferior compared to pressure washing. Manual sweeping (M) removed large debris from the brick surface, prevented the formation of a hydraulic barrier, and objectively played a pretreatment role. However, it would allow partial smaller particles to get inside the interior of the permeable brick, further blocking the permeable brick [26]. As a result, the recovery was less effective than vacuum suction or pressure washing, the average infiltration rate using M only increased by an average of 5.9%. Therefore, the order of the recovery effect is pressure washing (P) > vacuum suction (V) > manual sweeping (M). However, the inferior effect of V might also be related to the properties of permeable bricks. Previous research revealed that V was effective for PICP but ineffective for poured pavement PC and PA [15], and the effectiveness of V was dependent on the joint space, pavement age, and usage intensity [27]. In short, the maintenance efforts were dependent on multiple variables [28]. Therefore, it should be noted that the recovery effects were only adaptable to permeable brick in this study due to the different sensitivities of the permeable pavements to recovery measures.

For the recovery effect among the two-combination measures, manual cleaning together with pressure washing (MP), manual cleaning together with vacuum suction (MV), and pressure washing together with vacuum suction (PV) could recover 65.0%, 62.3%, and 69.3% for moderately clogged brick (C), respectively. The effect of MP and MV was little difference compared with P and V. For instance, the average permeability coefficient of P recovery was 0.0199 cm/s, however, the permeability coefficient of MP was only 0.0195, which is only 98.0% of the former. According to the correlation result calculated by correl function, the correlation between P and MP is close to 0.99, it represents that water pressure plays a major role in joint measures, while M hardly works. The average permeability coefficient of V recovery was 0.0184 cm/s, and the permeability coefficient of MV was 0.0187 cm/s, The effect is only improved by 1.0%. The correlation between V and MV is close to 0.91, it shows that V plays a major role and M plays an auxiliary role. This further suggested that, as a means of pretreatment, the effect of M was limited, and it might even be counterproductive in some cases. As some smaller particles entered inside the interior and blocked the brick, which hindered the effect of follow-up recovery measures, this might be the reason why the combined measures of MP and MV were not effective. PV had the higher efficiency than MP and MV, as the effects of P and V were superimposed and enhanced, some particles that were tightly bound to the brick could be loosened by P, further removed by V, which was the reason why the recovery effect of PV was better. Other studies [18,27,29] have also shown that a combination of PV had a better recovery effect in cleaning PC and PICP. But, for ceramic permeable brick, the correlation between P and PV was close to 0.98, it represents that water pressure played a major role in joint measures, and this difference might be related to the properties of these materials. It is speculated the sintering process made the brick structure more compact, the permeability coefficient of permeable brick was only 50–70 cm/h, which was significantly lower than 1000 cm/h of PICP and200 cm/h of PC [26,27], then the particles were relatively easier to accumulate on the surface, so as to make pressure washing more effective.

The permeability coefficient of MPV, which might be the best choice for combining three measures, could be restored to 73.0%. However, the recovery rate was only increased by 3.7% compared to PV. According to the correlation result calculated by correl function, the correlation between P and MPV is close to 0.93, while the correlation between M, V, and MPV being 0.91 and 0.83, it represents that water pressure also plays an important role in this joint measures, and other measures had a relatively auxiliary effect.

In our research, the effects of P, MP, PV, and MPV were 66.0%, 65.0%, 69.3%, and 73.0%, respectively. According to the calculation results of correl function, P plays a major role in the joint method, which reveals that the difference is very small comparing P, MP, PV and MPV. Therefore, P was recommended as the best recovery measure in brick clogging recovery, the joint methods was not recommended as the combined effect was not obvious and cost-effective.

### 3.2. Operating Conditions for Pressure Washing

High-pressure washing is the best technical measure for brick recovery; hence, it is necessary to study the operating conditions of high-pressure washing. In actual daily operating conditions, moisture conditions and the cleaning duration are important operating parameters that have an important influence on high-pressure hydraulic cleaning.

**Figure 6:**
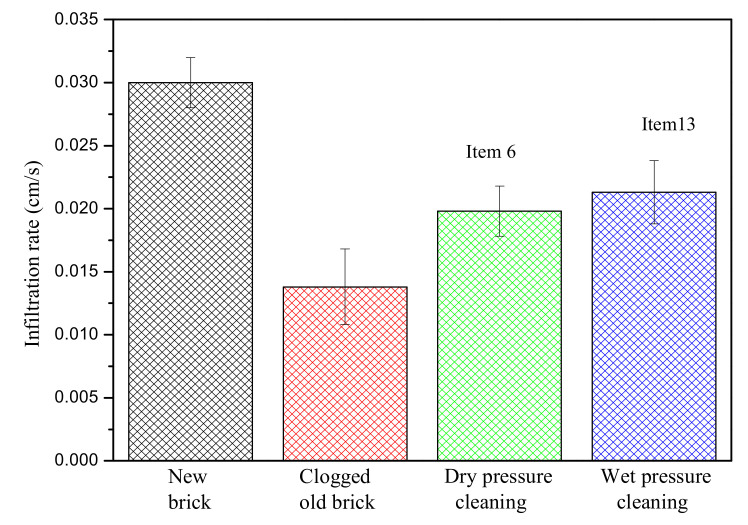
Recovery effect of pressure washing with different moisture statuses.

The recovery effect of pressure washing on clogged permeable bricks with dry and wet statuses is shown in Figure 6. The permeability coefficients of dry and wet permeable bricks after cleaning were restored to 66.0% and 71.0%, respectively, of the original permeability coefficient; obviously, the recovery effect was better in the wet state. The results show that the recovery effect can be improved by high-pressure cleaning immediately after clogging or rainfall. Clogged particles inside the pores of permeable bricks were in an unstable state and were easier to clean and remove in the wet state. Once the permeable brick was in a dry state, the clogged particles were consolidated in the pores and transformed into semipermanent or permanent blockage, making them more difficult to remove. These results are very instructive for actual cleaning operations, and a better recovery effect can be obtained by wetting permeable bricks before restoration or operating after rainfall.

**Figure 7:**
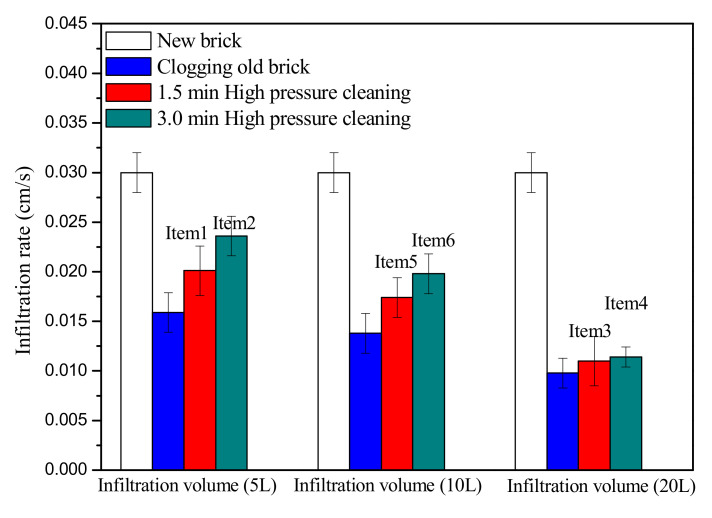
Recovery effect of pressure washing for different cleaning durations.

The recovery effect of pressure washing on different clogged permeable bricks with different cleaning durations is shown in Figure 7. Generally, the recovery effect improved with increasing cleaning duration. For permeable bricks with a low degree of clogging, the permeability coefficients were restored to 67.0% for a 1.5 min cleaning duration and 78.7% for a 3 min cleaning duration; therefore, the recovery efficiency increased by 12.7% when the cleaning time increased by 1.5 min. However, the recovery efficiency decreased with the aggravation of clogging, for permeable bricks with medium and high degrees of clogging, and when the washing time was increased from 1.5 min to 3 min, the recovery efficiencies were increased by only 8.0% and 1.3%, respectively, while the recovery efficiency was significantly reduced.

When the permeable brick was lightly blocked, the internal pores inside the brick were relatively large, while the degree of bonding between the particles and the brick was relatively poor. The particles that were not tightly combined were washed repeatedly by increasing the cleaning duration, and they could be washed away from the brick; thus, a good recovery effect was achieved. When the clogging was more serious, the particles and the brick were increasingly tightly combined, and a tight filter membrane was also formed between the particles, which undoubtedly increased the anti-impact load of pressure washing. Therefore, a stronger flushing force was needed to break the bottleneck caused by the filter membrane. The increase in cleaning time would not improve the shearing force acting on the filter membrane, and thus, the recovery effect on the permeability of permeable brick was not obvious.

Two strategies for improving the recovery effect of pressure washing can be obtained through this research. (1) Pressure washing should be conducted after rain or in wet conditions as often as possible; (2) It is necessary to increase the cleaning frequency or prolong the cleaning time in the case of no serious blockage.

### 3.3. Pore Change in the Recovery Process

**Figure 8:**
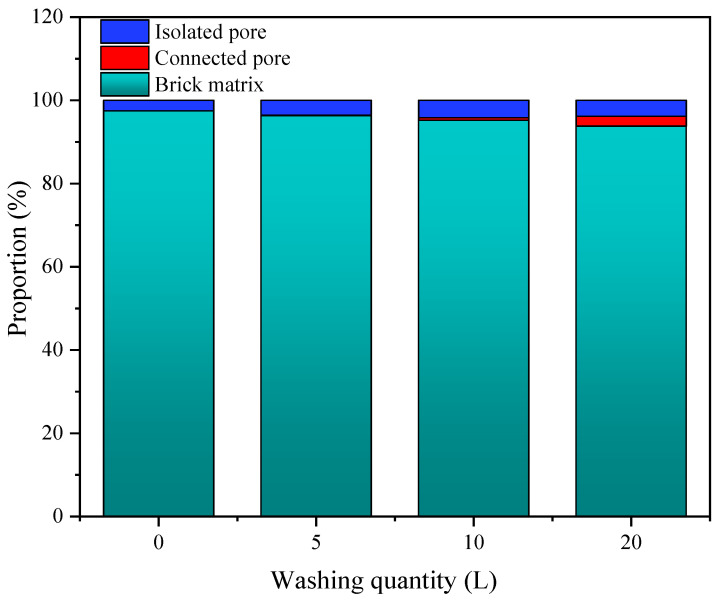
Analysis results based on 3D CT scanning images.

The analysis results based on 3D CT scanning images are shown in Figure 8. As presented in Figure 8, for the brick that had been basically clogged, the porosity of the brick increased with increasing flushing volume, and the proportion of the skeleton matrix decreased accordingly, indicating that a portion of the clogged pores were restored by hydraulic power. After cleaning with 20 L of water, the porosity increased from 0.025% to 0.062%, indicating a certain increase in pores. Since pores were the main channel through which rainwater passes, the permeability of the brick was significantly restored. Regarding pore morphology, usually, according to the size and position of the material pores, the pores can be divided into two types. One type is small in size, independent and unconnected, which is called isolated pores. The other type is larger and intersects and connects with each other, which is called connected pores [30]. In this research, the proportion of isolated pores increases to a certain extent as the amount of flushing water increases, and the proportion of connected pores increases faster, especially in the later stage of cleaning. Therefore, it is speculated that hydraulic cleaning has a process of first cleaning isolated pores and further connecting isolated pores to form connected pores.

## 4. Conclusions

In this study, ceramic permeable bricks were precisely clogged using a self-designed device to control the permeability coefficient. After clogging, different technical measures were adopted to restore the permeable brick. The effect and mechanism of pressure washing was investigated. Several conclusions can be drawn:

(1) The order of the recovery effect was pressure washing > vacuuming suction > manual sweeping. Pressure washing flushed out the surface and internal clogged particles with the flushing flow, while vacuuming could extract the particles that were not strongly adhered in the pores. However, as a means of pretreatment, manual sweeping prevented the formation of a hydraulic barrier but would allow partial smaller particles to further block the brick.
(2) Pressure washing was recommended the most appropriate recovery measure, the joint methods was not recommended as the combined effect was not obvious and poor performance to price ratio.
(3) Strategies improving the recovery effect of pressure washing could be obtained through this research. Increasing the frequency, prolonging the time, or cleaning under moist conditions can effectively improve the recovery effect of pressure washing.
(4) In the context of the recovery process, hydraulic cleaning can not only increase isolated pores but also connect the blocked particles, thus increasing the proportion of connected pores and dredges through water channels.

## Figures and Tables

**Table 1:** Recovery measures and effects of different permeable pavements.

Type of Pavement	Pavement Age/Year	Maintenance Type	Recovery Efficiency
PICP [15]	4	an Elgin Whirlwind vacuum truck with maximum power (2500 rpm)	Partial restoration of surface permeability with large spatial variability
PC [16]	2	Rinsing the surface with a garden hose or large hose	Over 90% of an area where permeability was obviously improved
		Vacuuming with a peak of 5.0 horsepower	20−80% of an area where permeability was improved
PA [17]	21	a Dustcontrol DC 50-W industrial wet/dry vacuum	Permeability coefficient of post-maintenance was 3.5 times higher than pre-maintenance
		a Nilfisk ALTO Poseidon 2–22 XT high pressure washer	Permeability coefficient of post-maintenance was 36 mm·min^−1^, 360 times higher than pre-maintenance
	28	a Dustcontrol DC 50-W industrial wet/dry vacuum	Permeability coefficient of post-maintenance was 6 times higher than pre-maintenance
		a Nilfisk ALTO Poseidon 2–22 XT high pressure washer	Permeability coefficient of post-maintenance was 0.42 mm·min^−1^, 4.2 times higher than pre-maintenance
PC [18]	6–18	A 4.85-kW vacuum sweeper	Permeability coefficient of post-maintenance was 25.4 cm·h^−1^, 10.45 times higher than pre-maintenance
		A 20.7-MPa pressure washer	Permeability coefficient of post-maintenance was 6 times higher than pre-maintenance
PC [19]	4	A 6.5 HP Briggs Stratton pressure washer	An average 20-fold infiltration rate improvement
PC [20]	1	Vacuum chamber driven with a 932.5 W pump.	Partial restoration while porosity recovered from 26% to 29%
	8	Vacuum chamber driven with a 932.5 W pump.	Less influenced while porosity recovered from 7.9% to 19.1%

**Table 2:** Experimental scheme of cleaning.

Item	Degree of Clogging	Cleaning Duration (min)	Maintenance Methods	Moisture Condition
1	Low	1.5	Pressure washing (P)	Dry
2	Low	3	Pressure washing (P)	Dry
3	High	1.5	Pressure washing (P)	Dry
4	High	3	Pressure washing (P)	Dry
5	Medium	1.5	Pressure washing (P)	Dry
6	Medium	3	Pressure washing (P)	Dry
7	Medium	3	Manual surface cleaning (M)	Dry
8	Medium	3	Vacuum cleaning (V)	Dry
9	Medium	3	Manual surface cleaning + Pressure washing (MP)	Dry
10	Medium	3	Manual surface cleaning + Vacuum cleaning (MV)	Dry
11	Medium	3	Pressure washing + Vacuum cleaning (PV)	Dry
12	Medium	3	Manual surface cleaning + Pressure washing + Vacuum cleaning (MPV)	Dry
13	Medium	3	Pressure washing(P)	Wet

## Data Availability

The data presented in this study are available on request from the corresponding author.
